# Supplementary material for: The Adult Congenital Heart Disease Anatomic and Physiological Classification: Associations with Clinical Outcomes in Patients with Atrial Arrhythmias
Source: Diagnostics (Basel). 2022 Feb 11;12(2):466. doi: 10.3390/diagnostics12020466 (PMC8870966; doi:10.3390/diagnostics12020466)
Supplement: Supplementary file 1 [file diagnostics-12-00466-s001.zip › diagnostics-1588635-supplementary.pdf]

Supplementary Appendix

The Adult Congenital Heart Disease Anatomic and Physiological Classification in Atrial Arrhythmias: Associations with Clinical Outcomes

This appendix has been provided by the authors to give readers additional information about their work.

Contents

**Table S1**	Clinical events definitions
**Table S2**	Criteria used for anatomic ACHD classification ACHD: I, II, or III
**Table S3**	Criteria used for physiological classification of ACHD: A, B, C, or D
**Table S4**	Incidence Rates and HRs for the Composite Outcome According to AnatC and PhyS
**Table S5**	Incidence Rates and Hazard Ratios for all-cause mortality, bleeding events and follow-up hospitalizations according to AnatC and PhyS
**Figure S1**	Prevalence of ACHD types in the study population
**Figure S2**	Visual distribution of mEHRA across AP-ACHD groups
**Figure S3**	Visual distribution of SF-36 across AP-ACHD groups

Table S1. Clinical events definitions

**Stroke**	Defined as a new-onset neurological dysfunction caused by central nervous system injury as a result of hemorrhage or infarction, of at least 24 hours duration (or if symptoms lasted <24 hours a clear matching lesion on CT or MRI), not due to another identifiable nonvascular cause (i.e., brain tumor, trauma).<br><br>All available data will be considered to support the diagnosis and sub-classification of the type of stroke. Strokes will be subclassified as “ischemic “or “primary hemorrhagic” based on imaging data, if available, or “undetermined cause” if imaging data are missing, according to the definitions below.<br><br>Ischemic stroke<br>• Ischemic stroke with no hemorrhage: stroke without intracerebral blood on brain imaging<br>• Ischemic stroke with hemorrhagic conversion: presence of blood felt to represent hemorrhagic conversion and not a primary hemorrhage<br>Primary hemorrhagic stroke<br>• A stroke with documentation on imaging (e.g., CT scan or MRI) of intracerebral, subdural or subarachnoid hemorrhage. Evidence of hemorrhagic stroke from other sources (lumbar puncture, neurosurgery, or autopsy) can also confirm the diagnosis.<br><br>Undetermined Stroke<br>• A stroke as a result of haemorrhage or infarction but with insufficient information classify as either ischemic or haemorrhagic.
**Systemic embolism**	A history consistent with an acute cessation of blood flow to a peripheral artery (or arteries) or evidence of embolism from other sources (eg. surgical specimens, angiography, vascular imaging) , localized to one of the following:

	• Lower or upper limb<br>• Intraocular<br>• Intra-abdominal viscera<br>• Other (to be specified)
**Pulmonary embolism**	Symptoms of PE with one of the following findings:<br>• A new intraluminal filling defect in (sub)segmental or more-proximal branches on CTPA, or in vessels more than 2.5 mm in diameter on the pulmonary angiogram.<br>• A new perfusion defect of at least 75% of a segment with a local normal ventilation result (high probability) on VQ scan.<br>• Inconclusive diagnosis of PE based on CTPA, pulmonary angiography, or VQ scan, but with demonstration of a new or recurrent DVT in the lower extremities by compression ultrasound or venography.
**Intracardiac thrombus**	Identified by echocardiography or cardiac MRI as a discrete echo-dense mass with well-defined borders that are distinct from the endocardium and seen throughout systole and diastole, in any of the 4 cardiac chambers (including atrial appendages)
**Major bleeding (ISTH)**	Defined as clinically overt bleeding that is associated with:<br>• A fall in hemoglobin of 2 g/dL or more<br>• A transfusion of ≥2 units of packed red blood cells or whole blood<br>• Bleeding in a critical site: intracranial, intraspinal, intraocular, pericardial, intra-articular, intramuscular with compartment syndrome, retroperitoneal<br>• Death (fatal bleeding)

**Clinically relevant nonmajor bleeding**	Defined as overt bleeding not meeting the criteria for major bleeding but associated with medical intervention, an unscheduled contact (visit or telephone call) with a physician, (temporary) cessation of study treatment, or discomfort for the patient, including:<br>• any bleeding compromising hemodynamics<br>• any bleeding leading to hospitalization<br>• subcutaneous (skin) hematoma if the size is >25 cm^2, or >100 cm^2 if provoked<br>• intramuscular hematoma<br>• epistaxis lasting for more than 5 minutes, if the episode was repetitive (i.e. two or more episodes of true bleeding, i.e. not spots on a handkerchief, within 24 hours), or led to an intervention (packing, electrocoagulation etc.)<br>• gingival bleeding if it occurred spontaneously (i.e. unrelated to tooth brushing or eating), or if it lasted for more than 5 minutes<br>• hematuria if it was macroscopic, and either spontaneous or lasting for more than 24 hours after instrumentation (e.g. catheter placement or surgery) of the urogenital tract<br>• macroscopic gastrointestinal hemorrhage: at least one episode of melena/hematemesis, if clinically apparent and hemoccult positive<br>• rectal blood loss, if more than a few spots on toilet paper<br>• hemoptysis, if more than a few speckles in the sputum and not occurring within the context of PE<br>• any other bleeding type that was considered to have clinical consequences for a patient
**Minor bleeding**	Defined as other overt bleeding events that do not fulfill the criteria of a major bleeding event or a clinically relevant nonmajor bleeding event (e.g., epistaxis that does not require medical attention).

**Transient ischemic attack**	Defined as new neurologic symptoms or deficit lasting less than 24 hours without acute infarction on CT or MRI (if available).
**Myocardial infarction (4th universal definition)**	Detection of a rise and/or fall of cardiac Troponin values with at least one value above the 99th percentile URL and with at least one of the following:<br>• Symptoms of acute myocardial ischemia<br>• New ischemic ECG changes<br>• Development of pathological Q waves<br>• Imaging evidence of new loss of viable myocardium or new regional wall motion abnormality in a pattern consistent with an ischemic etiology<br>• Identification of a coronary thrombus by angiographic methods or autopsy.
**Death**	Cause of death will be classified as follows:<br>Cardiovascular<br>• ischemic stroke<br>• hemorrhagic stroke<br>• systemic or pulmonary embolism<br>• other cardiovascular (i.e., myocardial infarction, sudden death, heart failure) and unobserved deaths<br>• unobserved deaths unless a non-cardiovascular cause can be clearly identified.<br>Non-cardiovascular<br>• bleeding

- other non-cardiovascular (i.e., malignancy, infection, trauma, pulmonary causes of death)
- Unknown death: Observed deaths of unknown cause

Intracranial bleeding that met the definition of stroke was included in both stroke and major bleeding endpoints.

CT, computer tomography; CTPA, computed tomography pulmonary angiography/angiogram; ISTH, international society on thrombosis and haemostasis; MI, myocardial infarction; MRI, magnetic resonance imaging; PE, pulmonary embolism; VQ scan; ventilation/perfusion lung scintigraphy

Table S2: Criteria used for anatomic ACHD classification: I, II, or III

Anatomic classification	Comments
**I (Simple Complexity)**	
Isolated small ASD	Per 2018 AHA/ACC Guidelines[1]; Isolated secundum ASD, excluding primum ASD and sinus venosus, with Qp:Qs < 1.5:1 and no chamber enlargement distal to the shunt
Isolated small VSD	Per 2018 AHA/ACC Guidelines[1]; Isolated VSD with Qp:Qs < 1.5:1 and no chamber enlargement distal to the shunt
Mild isolated pulmonic stenosis	Per 2018 AHA/ACC Guidelines[1]; Peak gradient < 36 mm Hg (peak velocity < 3 m/s) in transthoracic echocardiogram
Previously ligated or occluded ductus arteriosus	Per 2018 AHA/ACC Guidelines[1]
Repaired secundum ASD or sinus venosus defect without significant residual shunt or chamber enlargement	Per 2018 AHA/ACC Guidelines[1]; Significant shunt defined as ≥1.5:1; chamber enlargement defined as mild+
Repaired VSD without significant residual shunt or chamber enlargement	Per 2018 AHA/ACC Guidelines[1]; Significant shunt defined as ≥1.5:1; chamber enlargement defined as mild+
**II (Moderate Complexity)**	Per 2018 AHA/ACC Guidelines[1]
Aorto-left ventricular fistula	Per 2018 AHA/ACC Guidelines[1]
Anomalous pulmonary venous connection, partial or total	Per 2018 AHA/ACC Guidelines[1]
Anomalous coronary artery arising from the pulmonary artery	Per 2018 AHA/ACC Guidelines[1]
Anomalous aortic origin of a coronary artery from the opposite sinus	Per 2018 AHA/ACC Guidelines[1]
AVSD (partial or complete, including primum ASD)	Per 2018 AHA/ACC Guidelines[1]
Congenital aortic valve disease	Per 2018 AHA/ACC Guidelines[1]
Congenital mitral valve disease	Per 2018 AHA/ACC Guidelines[1]; Excluding mitral valve prolapse
Coarctation of the aorta	Per 2018 AHA/ACC Guidelines[1]
Ebstein anomaly (mild, moderate, and severe)	Per 2018 AHA/ACC Guidelines[1]
Infundibular right ventricular outflow obstruction	Per 2018 AHA/ACC Guidelines[1]
Ostium primum ASD	Per 2018 AHA/ACC Guidelines[1]

Moderate and large unrepaired secundum ASD	Per 2018 AHA/ACC Guidelines[1]
Moderate and large persistently patent ductus arteriosus	Per 2018 AHA/ACC Guidelines[1]
Pulmonary valve regurgitation (moderate or greater)	Per 2018 AHA/ACC Guidelines[1]
Pulmonary valve stenosis (moderate or greater)	Per 2018 AHA/ACC Guidelines[1]; Peak gradient ≥ 36mmHg (velocity ≥3 m/s) or subjectively ≥ moderate
Peripheral pulmonary stenosis	Per 2018 AHA/ACC Guidelines[1]
Sinus of Valsalva fistula/aneurysm	Per 2018 AHA/ACC Guidelines[1]
Sinus venosus defect	Per 2018 AHA/ACC Guidelines[1]
Subvalvar aortic stenosis (excluding HCM)	Per 2018 AHA/ACC Guidelines[1]
Supravalvar aortic stenosis	Per 2018 AHA/ACC Guidelines[1]
Straddling AV valve	Per 2018 AHA/ACC Guidelines[1]
Repaired tetralogy of Fallot (toF)	Per 2018 AHA/ACC Guidelines[1]; Repaired toF, without pulmonary atresia
VSD with associated abnormality and/or moderate or greater shunt	Per 2018 AHA/ACC Guidelines[1]; VSD & ≥ moderate shunt (Qp:Qs≥1.5:1) or chamber enlargement
**III (Great Complexity)**	Per 2018 AHA/ACC Guidelines[1]
Cyanotic congenital heart defect (unrepaired or palliated, all forms)	Per 2018 AHA/ACC Guidelines[1]
Double-outlet ventricle	Per 2018 AHA/ACC Guidelines[1]
Fontan procedure	Per 2018 AHA/ACC Guidelines[1]
Interrupted aortic arch	Per 2018 AHA/ACC Guidelines[1]
Mitral atresia	Per 2018 AHA/ACC Guidelines[1]
Single ventricle (including double inlet left ventricle, tricuspid atresia, hypoplastic left heart, any other anatomic abnormality with a functionally single ventricle)	Per 2018 AHA/ACC Guidelines[1]
Pulmonary atresia (all forms)	Per 2018 AHA/ACC Guidelines[1]
TGA (classic or d-TGA; CCTGA or l-TGA)	Per 2018 AHA/ACC Guidelines[1]
Truncus arteriosus	Per 2018 AHA/ACC Guidelines[1]; Repaired or unrepaired PA/IVS or toF/PA

[1]2018 AHA/ACC Guideline for the Management of Adults With Congenital Heart Disease: A Report of the American College of Cardiology/American Heart Association Task Force on Clinical Practice Guidelines. J Am Coll Cardiol 2018;Aug 16

*ACHD; adult congenital heart disease, ASD; atrial septal defect, VSD = ventricular septal defect, TTE = trans-thoracic echocardiograph, AV; atrio-ventricular, AVSD; atrioventricular septal defect, IVS; intact ventricular septum, PA; pulmonary atresia, PS; pulmonary stenosis, RVOT = right ventricular outflow tract, toF = tetralogy of Fallot, TGA = transposition of the great arteries (cc = congenitally/physiologically corrected)

Table S3: Criteria used for physiological classification of ACHD: A, B, C, or D

Physiological classification	Diagnostic criteria used
**Stage A**	
• NYHA class I	No symptoms and no limitation in ordinary physical activity, e.g. shortness of breath when walking, climbing stairs etc
• No arrhythmia	
• Normal exercise capacity	
• No hemodynamic or anatomic sequelae	
• Normal renal, hepatic, and pulmonary function	
**Stage B**	
• NYHA class II	Mild symptoms (mild shortness of breath and/or angina) and slight limitation during ordinary activity
• Mild hemodynamic sequelae (aortic enlargement, ventricular enlargement, ventricular dysfunction)	Mild sub-pulmonary/sub-systemic ventricular dysfunction (LVEF 40-50% and/or RVEF 35-45%); Mild aortic enlargement (maximum diameter 3.5-3.9 cm), mild sub-systemic/sub-pulmonary ventricular enlargement
• Mild valvular disease	Mild aortic stenosis/ aortic regurgitation/ mitral stenosis/ mitral regurgitation; not including pulmonary or tricuspid as these can be found in healthy patients.
• Trivial or small shunt (not hemodynamically significant)	No evidence of chamber enlargement distal to the shunt
• Arrhythmia not requiring treatment	Clinically relevant arrhythmia in prior 24 months not treated with medication
• Abnormal objective cardiac limitation to exercise	Peak VO_2<85% of the mean value for that diagnostic group[1]
**Stage C**	

• NYHA class III	Marked limitation in activity due to symptoms, even during less-than-ordinary activity, e.g. walking short distances (20—100 m).Comfortable only at rest
• Significant (≥ moderate) valvular disease; ≥moderate ventricular dysfunction (systemic and/or sub-pulmonic)	≥Moderate grade of any valve dysfunction; ≥moderate reduction in sub-systemic or sub-pulmonary ventricular function (LVEF < 40% and/or RVEF <35%)
• Moderate aortic enlargement	Moderate aortic enlargement (maximum diameter 4.0-4.9cm)
• Venous or arterial stenosis	Re-coarctation after CoA repair/ supravalvular aortic obstruction/ venous baffle obstruction/ supravalvular pulmonary stenosis/ branch PA stenosis/ stenosis of cavo-pulmonary connection/ pulmonary vein stenosis
• Mild or moderate hypoxemia/cyanosis	Oxygen saturation measured by pulse oximetry ≤ 90%
• Hemodynamically significant shunt	Evidence of chamber enlargement distal to shunt and/ or evidence of sustained Qp:Qs ≥1.5:1
• Arrhythmias controlled with treatment	Clinically relevant arrhythmia in prior 24 months treated with medication, catheter, ICD, or pacemaker
• Pulmonary hypertension (less than severe)	Mean PA pressure by right heart catheterization ≥25 mmHg.
• End-organ dysfunction responsive to therapy	eGFR 30-60, moderate restrictive lung disease (FVC 50-70% predicted), cirrhosis with albumin concentration ≥ 3 g/dL or MELD-XI score ≤ 12[2]
**Stage D**	
• NYHA class IV	Severe limitations. Experiences symptoms even while at rest. Mostly bedbound patients
• Severe aortic enlargement	Severe aortic enlargement (maximum diameter ≥ 5.0cm)
• Arrhythmia refractory to treatment	Clinically relevant arrhythmia including symptomatic bradyarrhythmia and atrial or ventricular tachyarrhythmias, in the prior 24 months with continued arrhythmia despite medical therapy, catheter ablation, or cardioversion
• Severe hypoxemia (almost always associated with cyanosis)	Severe hypoxemia (oxygen saturation at rest <85%)

• Severe pulmonary hypertension	Right heart catheterization: mean pulmonary arterial pressure ≥35 mm Hg, and/or treatment for PH
• Eisenmenger syndrome	Right-to-left shunt (Qp:Qs <1) and elevated pulmonary pressures
• Refractory end-organ dysfunction	On dialysis, eGFR<30,4 severe restrictive lung disease (FVC <50%), cirrhosis with albumin <3 and/or MELD-XI score >12

*ACHD; adult congenital heart disease, LVEF; left ventricular ejection fraction; RVEF; right ventricular ejection fraction; QP:QS = pulmonary flow:systemic flow, ICD; implantable cardioverter-defibrillator, PA; pulmonary artery, eGFR; estimated glomerular filtration rate, FVC; forced vital capacity

Table S4. Incidence Rates and HRs for the Composite Outcome According to AnatC and PhyS

	**AnatC**				**PhyS**			
	**I (n=55)**	**II (n=56)**	**III (n=46)**	**p-value for trend**	**B (n=93)**	**C (n=58)**	**D (n=6)**	**p-value for trend**
Events (n=47)*	18 (32.7%)	19 (33.9%)	10 (21.7%)	-	23 (24.7%)	22 (37.9%)	2 (33.3%)	-
Incidence rate** (95% CI)	24 (15-38)	24 (15-37)	14 (7-26)	0.598	18 (11-26)	26 (17-39)	33 (6-110)	0.150
Unadjusted HR (95% CI)	1	1.02 (0.54-1.95)	0.83 (0.40-1.72)	0.639	1	1.46 (0.81-2.62)	1.80 (0.42-7.63)	0.175
Adjusted HR*** (95% CI)	1	1.12 (0.37-3.41)	1.06 (0.24-4.63)	0.980	1	1.79 (0.69-4.67)	8.15 (1.52-43.59)	**0.040**

*Events correspond to the composite outcome of mortality, any major thrombotic event, any major or clinically relevant non-major bleeding event, or hospitalization for any cause
**per 100 patient years
***model adjusted for physiological and anatomic classification, age, CHA2DS2-VASc and HAS-BLED scores and left atrial diameter
CHA2DS2-Vasc (congestive heart failure, hypertension, age ≥ 75 years, diabetes mellitus, stroke/transient ischemic attack, vascular disease, age 65 to 74 years, sex category); HAS-BLED (Hypertension, Abnormal renal/ liver function, Stroke, Bleeding history or predisposition, Labile INR, Elderly, Drugs/alcohol); CI, confidence interval; HR, hazard ratio

Table S5. Incidence Rates and Hazard Ratios for all-cause mortality, bleeding events and follow-up hospitalizations according to AnatC and PhyS

*per 100 patient years

	AnatC				PhyS			
	I (n=55)	II (n=56)	III (n=46)	**p-value for trend**	B (n=93)	C (n=58)	D (n=6)	**p-value for trend**
**All-cause mortality (n=6)**	2 (3.6%)	4 (7.1%)	0 (0%)	-	4 (4.3%)	2 (3.4%)	0 (0%)	-
Incidence rate* (95% CI)	2.7 (0.5-9)	5.1 (1.6-12.4)	-	0.388	3.1 (1-7.4)	2.4 (0.4-7.8)	-	0.622
Unadjusted HR (95% CI)	1	1.4 (0.23-8.21)	-	0.639	1	0.97 (0.16-5.78)	-	0.877
**major or clinically relevant non-major bleeding events (n=22)**	9 (16.4%)	7 (12.5%)	6 (13%)	-	10 (10.8%)	11 (19%)	1 (16.6%)	-
Incidence rate* (95% CI)	12 (6-22)	9 (3.9-17.8)	8.7 (3.5-18.1)	0.616	7.7 (3.9-13.7)	12.9 (6.8-22.5)	16 (0.8-82)	0.198
Unadjusted HR (95% CI)	1	0.75 (0.28-2.02)	0.76 (0.27-2.13)	0.812	1	1.70 (0.72-4.01)	1.93 (0.25-15.15)	0.443
**Hospitalizations (n=20)**	7 (12.7%)	9 (16.1%)	4 (8.7%)	-	9 (9.7%)	9 (15.5%)	2 (33.3%)	**-**
Incidence rate* (95% CI)	9.5 (4.1-18.7)	11.5 (5.6-21.2)	5.8 (1.8-14)	0.578	6.9 (3.4-12.7)	10.6 (5.2-19.4)	33.3 (5.6-110)	0.278
Unadjusted HR (95% CI)	1	1.20 (0.45-3.22)	0.64 (0.19-2.19)	0.556	1	1.54 (0.61-3.88)	2.16 (0.27-17.06)	0.578

CI, confidence interval

Figure S1: Prevalence of ACHD types in the study population

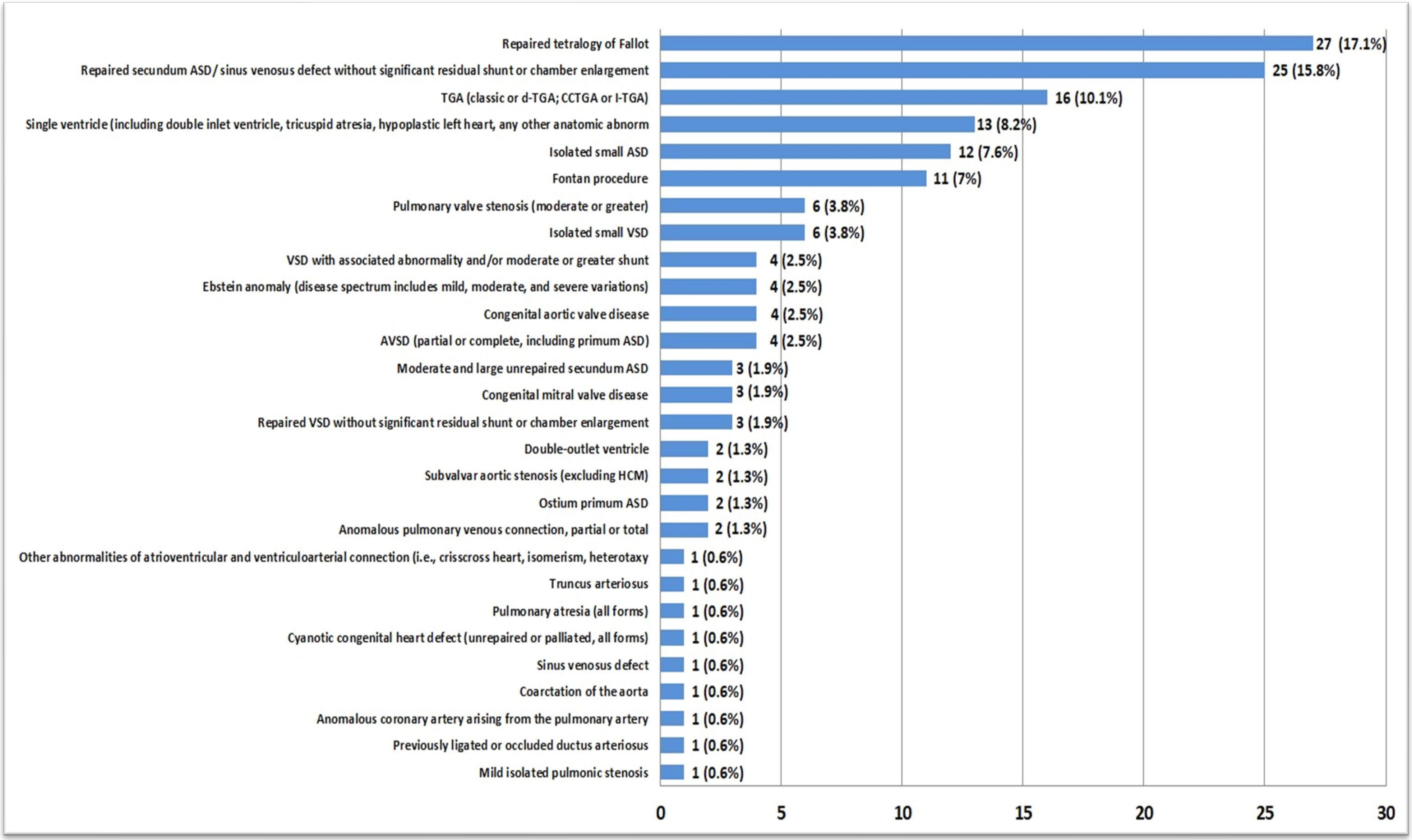

Figure S2. Visual distribution of mEHRA across AP-ACHD groups

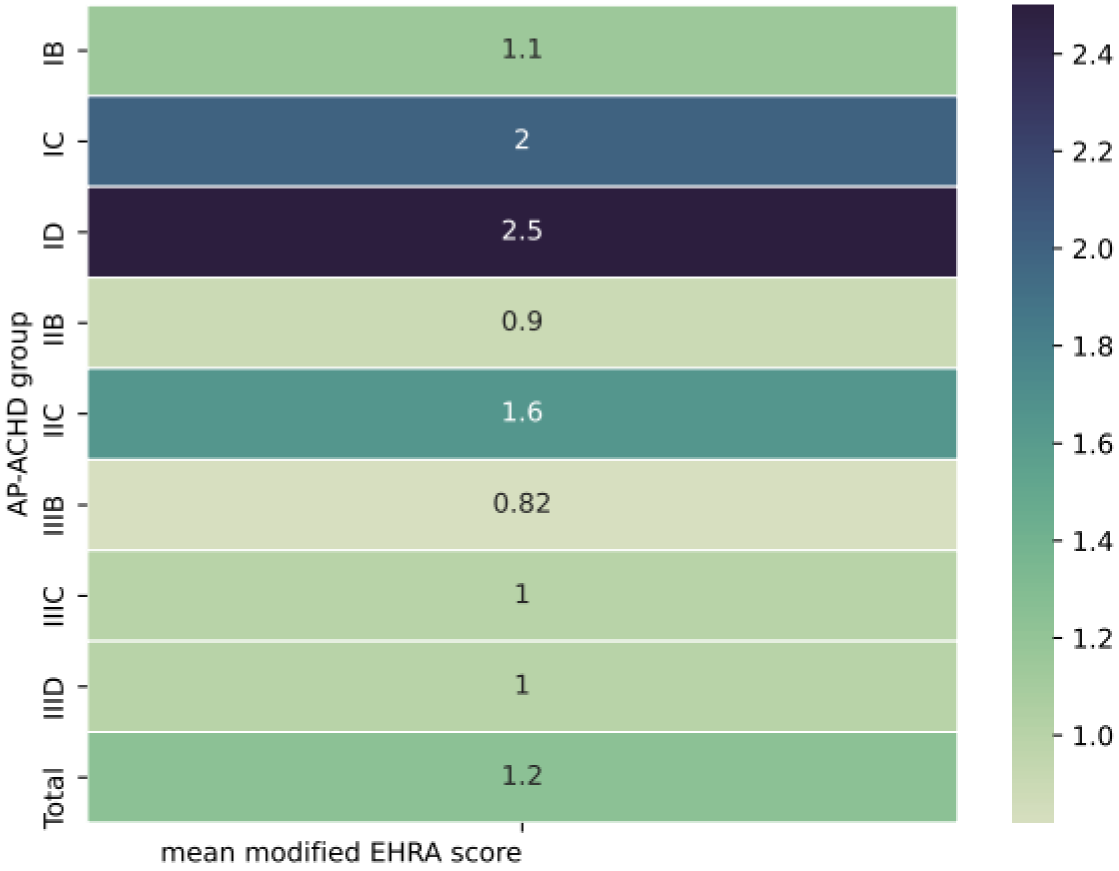

AP-ACHD, anatomic and physiological classification of adult congenital heart disease; mEHRA, modified European Heart Rhythm Association

Figure S3. Visual distribution of SF-36 across AP-ACHD groups

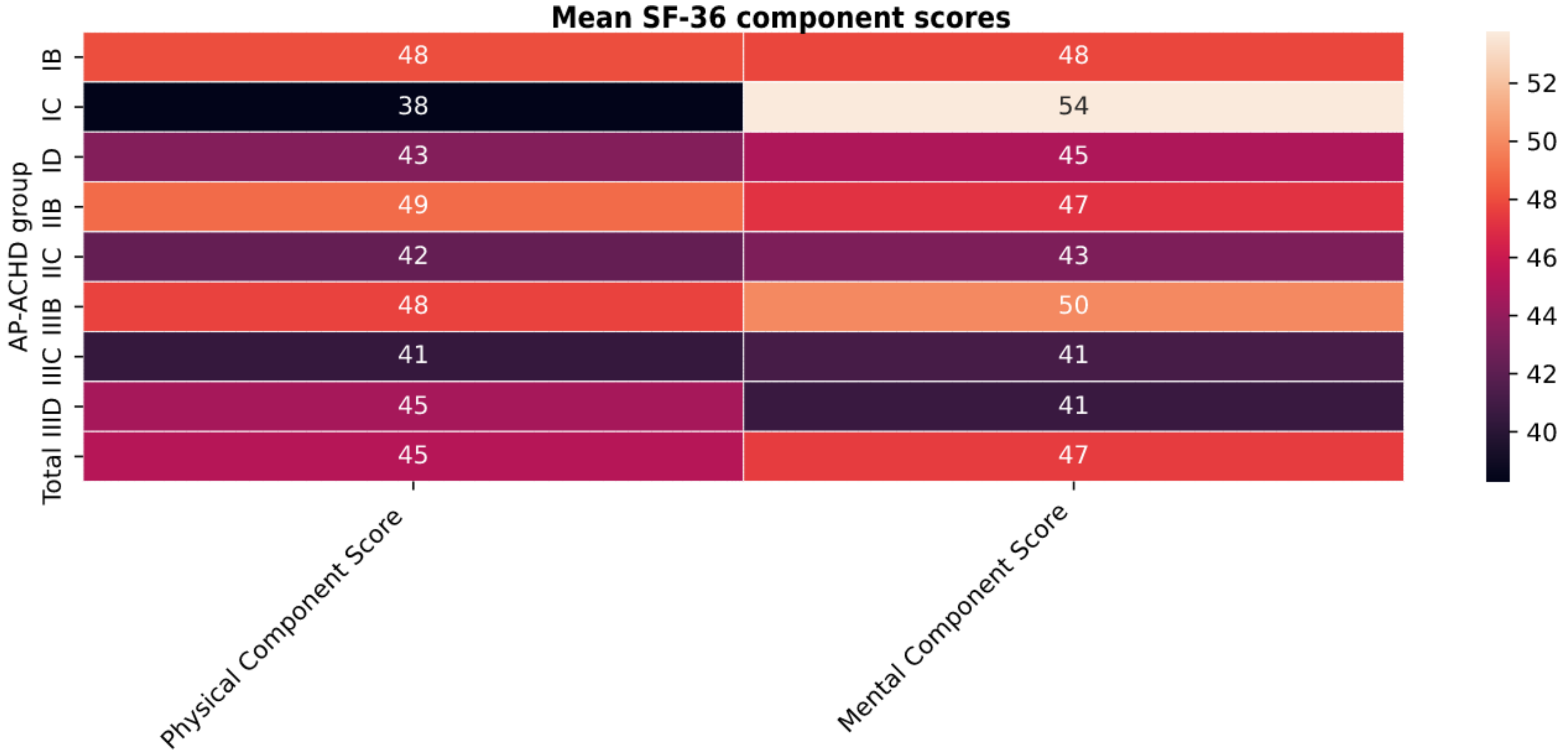

AP-ACHD, anatomic and physiological classification of adult congenital heart disease; SF-36, Short Form-36

REFERENCES

1. Kempny A, Dimopoulos K, Uebing A, et al. Reference values for exercise limitations among adults with congenital heart disease. Relation to activities of daily life—single centre experience and review of published data. *Eur Heart J*. 2012;33(11):1386-1396. doi:10.1093/eurheartj/ehr461
2. Murata M, Kato TS, Kuwaki K, Yamamoto T, Dohi S, Amano A. Preoperative hepatic dysfunction could predict postoperative mortality and morbidity in patients undergoing cardiac surgery: Utilization of the MELD scoring system. *Int J Cardiol*. 2016;203:682-689. doi:10.1016/j.ijcard.2015.10.181
3. Wernly B, Lichtenauer M, Franz M, et al. Model for End-stage Liver Disease excluding INR (MELD-XI) score in critically ill patients: Easily available and of prognostic relevance. *PLoS One*. 2017;12(2). doi:10.1371/journal.pone.0170987
4. Dimopoulos K, Diller G-P, Koltsida E, et al. Prevalence, predictors, and prognostic value of renal dysfunction in adults with congenital heart disease. *Circulation*. 2008;117(18):2320-2328. doi:10.1161/CIRCULATIONAHA.107.734921
5. Camm AJ, Savelieva I, Lip GYH. Rate control in the medical management of atrial fibrillation. *Heart*. 2007;93(1):35-38. doi:10.1136/hrt.2006.099903
